# Occurrence of multidrug-resistant *Mycobacterium tuberculosis* in upper Southern Thailand

**DOI:** 10.14202/vetworld.2024.1405-1412

**Published:** 2024-06-28

**Authors:** Pathom Karaipoom, Phirabhat Saengsawang, Arisa Bromnavej, Supattra Sangsong, Pinkamon Waseewiwat, Bunrit Bunsanong, Veeranoot Nissapatorn, Maria de Lourdes Pereira, Watcharapong Mitsuwan

**Affiliations:** 1 Office of Disease Prevention and Control Region 11^st^, Nakhon Si Thammarat, 80000, Thailand; 2 Akkhraratchakumari Veterinary College, Walailak University, Nakhon Si Thammarat, 80160, Thailand; 3 One Health Research Center, Walailak University, Nakhon Si Thammarat 80160, Thailand; 4 School of Allied Health Sciences, Southeast Asia Water Team, World Union for Herbal Drug Discovery, and Research Excellence Center for Innovation and Health Products, Walailak University, Nakhon Si Thammarat, Thailand; 5 CICECO-Aveiro Institute of Materials and Department of Medical Sciences, University of Aveiro, Aveiro, Portugal; 6 Center of Excellence in Innovation of Essential Oil and Bioactive Compounds, Walailak University, Nakhon Si Thammarat, Thailand

**Keywords:** *inhA* and *katG* genes, isoniazid, multi-drug resistance, *Mycobacterium tuberculosis*, upper Southern of Thailand

## Abstract

**Background and Aim:**

*Mycobacterium tuberculosis* causes global concern with tuberculosis (TB). Multidrug-resistant TB (MDR-TB) and extensively drug-resistant TB (XDR-TB) pose additional challenges, as they resist to multiple first-line drugs. This study investigated the occurrence of TB, antibiotic resistance due to *inhA* and *katG* gene mutations, and multidrug resistance in *M. tuberculosis* during fiscal years 2020–2022.

**Materials and Methods:**

Samples were gathered from hospitals in seven provinces of upper Southern Thailand. The study investigated the correlation between *inhA* and *katG* gene mutations in *M. tuberculosis* and the development of antimicrobial resistance and isoniazid resistance.

**Results:**

A total of 19,186 samples were sent to the Office of Disease Prevention and Control Region 11^st^, Nakhon Si Thammarat, Thailand. The results showed that 51% of the samples were obtained from patients located in Nakhon Si Thammarat, followed by Surat Thani provinces. Regarding the spatial distribution of TB-infected cases, the incidence of TB was high in the province, which has a moderate to high population density. The highest average occurrence of TB in this study was found in Phuket province (9.75/100,000 risk person-year). The detected isoniazid resistance was 394, 255, and 179 cases in 2020, 2021, and 2022, respectively. A total of 99 isolates were MDR, whereas four isolates were XDR. The antimicrobial resistance associated with the *inhA* mutation was 192, 142, and 105 isolates, respectively, whereas the resistance associated with the *katG* mutation was 249, 182, and 120 cases in 2020, 2021, and 2022, respectively.

**Conclusion:**

These findings contribute to the understanding of the occurrence of antibiotic-resistant TB that could lead to use as data for preventing MDR-TB.

## Introduction

*Mycobacterium tuberculosis* infections continue to pose a major global health issue, marked by high morbidity and mortality rates. Recently, 10.6 million new tuberculosis (TB) cases were reported by the World Health Organization (WHO) in 2021 [1]. In many organs, including the lung, lymph nodes, and bone, TB are primarily caused by the pathogen. *M. tuberculosis* infections result in diverse clinical manifestations, ranging from inapparent to active pulmonary or extrapulmonary (EP) disease. Pulmonary TB is the most common clinical form of the illness and is responsible for the majority of TB cases that have been documented globally [2]. EP TB, a form of *M. tuberculosis* infection outside the lungs, is a significant cause of morbidity and mortality in humans [2].

Multidrug-resistant TB (MDR-TB) results from *M. tuberculosis* strains that resist treatment with isoniazid and rifampicin, two primary anti-TB medications [3]. TB infection is treated with first-line drugs isoniazid and rifampicin. The combination of isoniazid, rifampicin, and pyrazinamide effectively cures *M. tuberculosis* strains for 3 months that are fully drug-susceptible [4]. Failure to comply with treatment or take adequate medication contributes to the development and spread of drug resistance in *M. tuberculosis* against all relevant antibiotics [5]. Therefore, MDR-TB is a TB caused by *M. tuberculosis* that is resistant to treatment with at least two of the first-line anti-TB drugs, including isoniazid and rifampicin [3]. Furthermore, extensively drug-resistant TB (XDR-TB) is defined as MDR-TB plus resistance to one of the fluoroquinolones and to one of the second-line injectable anti-TB drugs: amikacin, kanamycin, or capreomycin [6].

Isoniazid is a commonly used drug for treating *M. tuberculosis* infections by inhibiting NADH-dependent enoyl-ACP reductase encoded by *inhA* and prevents mycolic acid synthesis [7]. Isoniazid acts as a prodrug that requires activation by the *M. tuberculosis* catalase-peroxidase enzyme *katG*. Mutations in *katG* and *inhA* genes confer resistance to isoniazid [7]. Infection control is becoming more challenging because of rising drug resistance, especially to the first-line antibiotic isoniazid, and the occurrence of MDR-TB [8].

This study aimed to investigate the occurrence and antibiotic resistance of TB caused by mutations in the *inhA* and *katG* genes in *M. tuberculosis* from patients in upper Southern Thailand (2020–2022). Chumphon, Surat Thani, Nakhon Si Thammarat, Phuket, Krabi, Phang Nga, and Ranong are the seven provinces located in this area.

## Materials and Methods

### Ethical approval

A retrospective dataset of TB during the fiscal years 2020–2022 was received from the Office of Disease Prevention and Control Region 11^st^, Nakhon Si Thammarat, Thailand. The laboratory data were secondary data from a TB laboratory performed at the Office of Disease Prevention and Control Region 11^st^, Nakhon Si Thammarat, Thailand, under the Ministry of Public Health Thailand. All study procedures were approved by the Ethics Scientific Committee of Boromarajonani College of Nursing, Nakhon Si Thammarat, Thailand (Ref No. Exc-10/2566).

### Study period, location, and sample collection

**Figure-1:**
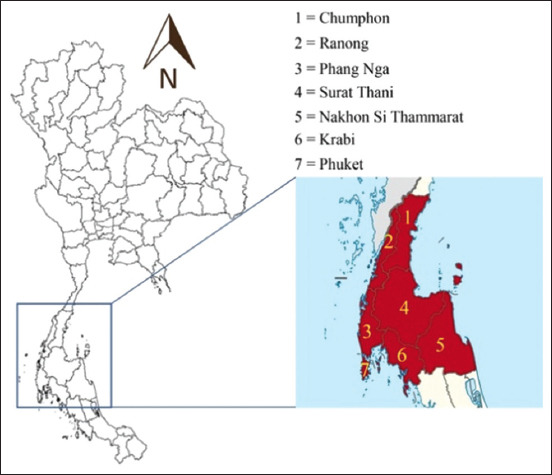
Locations in upper Southern Thailand. All samples of Tuberculosis from this area are sent to Office of Disease Prevention and Control Region 11^st^, Nakhon Si Thammarat, Thailand. [Source of the map: https://cleverlearn-hocthongminh.edu.vn].

The study was conducted from August 2023 to February 2024. All data in this study were a secondary data in fiscal years 2020–2022. In Thailand, the fiscal year runs from 1^st^ October to 30^th^ September of the following year. Therefore, fiscal year 2020 spanned from October 1^st^, 2019 to September 30^th^, 2020. The approach for fiscal years 2021 and 2022 was carried out as previously explained. The Office of Disease Prevention and Control Region 11^st^ collected the clinical samples associated with TB from hospitals located in seven provinces, including Nakhon Si Thammarat, Surat Thani, Phuket, Krabi, Chumphon, Ranong, and Phang Nga provinces (Figure-1). The collected samples are shown in Table-1.

**Table-1:** Sample collection.

Samples	Numbers of samples (n)/Year			Total (n)

	2020	2021	2022	
Abdominal fluid	7	1	-	8
Ascitic fluid	2	1	-	3
Bronchoalveolar lavage	64	-	2	66
Blood	34	11	2	47
Bone marrow	19	1	-	20
Bone/Joint	5	1	-	6
Bronchoalveolar lavage	67	-	4	71
Cerebrospinal fluid	64	5	-	69
Gastric lavage	9	2	-	11
Gastric washing	6	-	-	6
Joint fluid	5	-	1	6
Lymph node	27	1	-	28
Peritoneal dialysis fluid	8	-	-	8
Pericardial fluid	9	1	1	11
Peritoneal fluid	7	-	-	7
Pleural effusion	1	-	-	1
Pleural fluid	93	13	9	115
Pus	58	14	6	78
Skin	1	-	-	1
Sputum	6,954	7,053	4,481	18,488
Stool	8	-	-	8
Suction tube (sputum)	5	1	1	7
Synovial fluid	2	-	-	2
Tissue	39	3	1	43
Urine	45	2	-	47
Other	26	-	3	29
Total	7,565	7,110	4,511	19,186

### Detection, culture, and identification of *M. tuberculosis*

**Figure-2:**
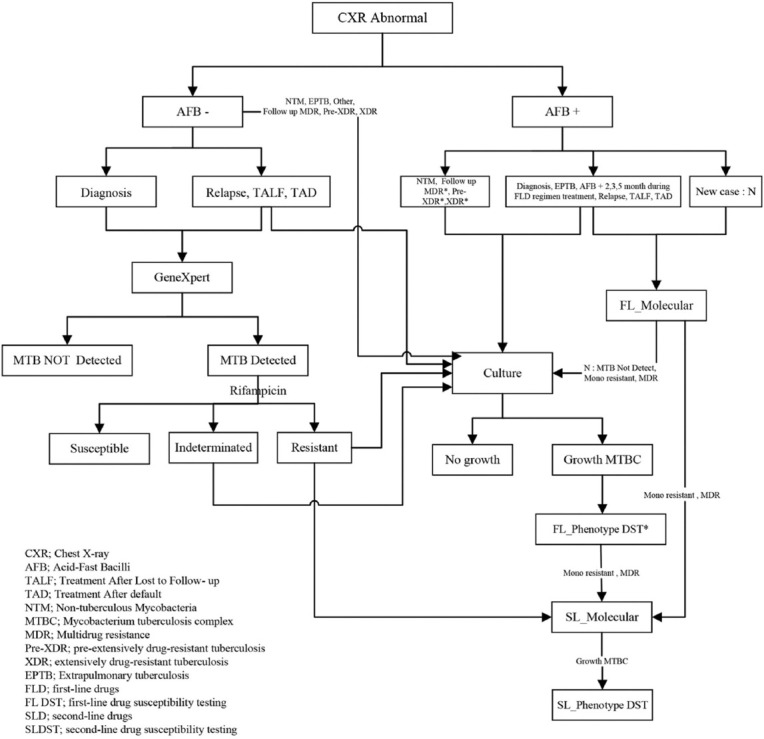
Flow chart of the detection of *Mycobacterium tuberculosis* and antibiotic susceptibility from the samples submitted to Office of Disease Prevention and Control Region 11^st^, Nakhon Si Thammarat, Thailand.

The detection, culture, and identification of *M. tuberculosis* from the collected samples were performed as shown in the overall flowchart of this study in Figure-2. Patients with abnormal X-ray chests were screened for the presence of acid-fast bacilli (AFB) in the clinical samples. For AFB-positive, the samples were recorded according to the criteria as non-*M. tuberculosis* (NTM, *Mycobacterium* spp. without *M. tuberculosis*), followed up, multidrug resistance, pre-XDR, XDR, diagnosis, EP, followed up on the treatments, relapse, treatment after loss failure (TALF), treatment after default (TAD), and new case. All the samples as described above, except the new case, which is non-resistant to isoniazid and rifampicin, were then cultured in Middlebrook 7H9 liquid media associated with BACTEC™ MGIT™ 960 (MGIT 960) (Becton Dickinson, Shannon, Ireland) and Lowenstein-Jensen medium (Nonthaburi, Thailand) and incubated at 37 ± 1°C for 6–8 weeks. The bacteria were identified as *M. tuberculosis* using a BD BACTEC™ MGIT™ and BD MGIT™ TBc identification test (TAUNS Laboratory, Japan). For negative AFB, samples were recorded according to the criteria described above. The samples, including NTM, EP, follow-up, MDR, pre-XDR, and XDR, were cultured as described above, while the samples groups of diagnosis, relapse, TALF, and TAD were further detected for the presence of the *M. tuberculosis* genome by GeneXpert (Cepheid, Solna, Sweden).

### Antimicrobial susceptibility

The antibiotic susceptibility of *M. tuberculosis* clinical isolates was investigated using MGIT 960 (Becton Dickinson, Shannon, Ireland). The antibiotics primarily contain isoniazid, rifampicin, ethambutol, and streptomycin. The bacterial colonies were mixed in tubes containing glass beads. Then, the sample was adjusted for turbidity to the 0.5 MacFarland standard. The samples were then diluted in sterile water (1:100 and 1:5). The sample at 1:100 was added to the tube containing the antibiotics to investigate antibiotic susceptibility. It was noticed that the bacterial suspension at 1:5 was used as the growth control because it could present the intense luminescence that presented growth-positive results. The samples were incubated at 37 ± 1°C for13 days. The results were interpreted as sensitive (S), resistant (R), and invalid (I). In addition, the sample tubes that were contaminated with other organisms were interpreted as contaminated (C). MDR is defined as resistance to at least two of the first-line anti-TB drugs, including isoniazid and rifampicin. XDR was defined as MDR in the presence of the antibiotic resistance genes *gryrA* or *gryrB* (fluroquinolone) and *res* or *eis* (aminoglycoside). In addition, pre-XDR was defined as MDR with resistance plus at least fluoroquinolone or second-line injectables.

### Detection of mutations associated with *inhA* and *katG* genes

All antibiotic-resistant *M. tuberculosis* mutations associated with *inhA* and *katG* genes were then detected by Line probe assay (Genotype® MTBDRplusVer2.0 [HainLife Science, Nehren, Germany]) and real-time polymerase chain reaction (PCR) as previously described by WHO [6]. RNA was extracted using a commercial Qiagen kit (Nehren, Germany). Only positive cultures (MGIT 960, LJ Medium) or AFB-positive samples were used for the test. Following the manufacturer’s instructions, the process comprised DNA extraction, multiplex amplification using biotinylated primers, and DNA reverse hybridization. Mutations in the *inhA* regulatory area and *katG* gene, which confer resistance, were evaluated.

### Statistical and spatial analyses

The collected data were analyzed using descriptive statistics. All statistical analyses were performed using the R programming language version 4.3.2 (https://www.r-project.org). In addition, data on population demography and the number of TB-infected cases were used to calculate the incidence classified by years and area. The calculation was performed using Microsoft Excel 2021, and the incidence was presented as per 100,000 risk person-years.

Information on TB incidence was used to create the spatial distribution map using Quantum GIS desktop 3.22 software (https://www.qgis.org). The grading of incidence per area followed the Jenks Natural Break method with five classes. The classes of incidence per area were classified as none, low incidence, moderate incidence, high incidence, and extremely high incidence.

## Results

### Sample collection and identification of *M. tuberculosis*

**Figure-3:**
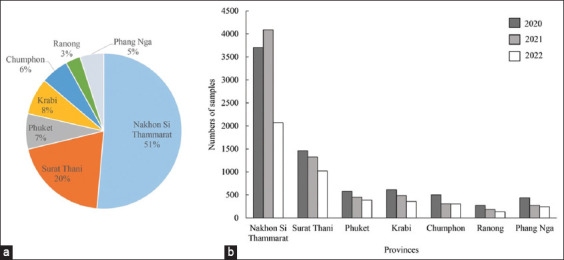
(a) Distribution of samples associated with Tuberculosis in different area controlled by Office of Disease Prevention and Control Region 11st, Nakhon Si Thammarat, Thailand. (b) Numbers of the samples in different areas in the fiscal years 2020-2022.

Samples were collected from patients who were suspected of having TB as diagnosed by doctors and other medical diagnoses. A total of 19,186 samples associated with TB were sent to the Office of Disease Prevention and Control Region 11^st^, Nakhon Si Thammarat, Thailand. The results revealed that 51% of the samples were received from patients located in Nakhon Si Thammarat province, followed by Surat Thani province (20%) and Krabi province (8%), respectively (Figure-3a). During the years 2020–2022, the number of samples associated with TB decreased over time (Table-1 and Figure-3b). However, the total number of samples in Nakhon Si Thammarat province in 2021 was higher than those in 2020 and 2022. It was noticed that the highest number of samples collected from the patients was sputum, followed by pleural fluid and pus.

### Spatial analyses

**Figure-4:**
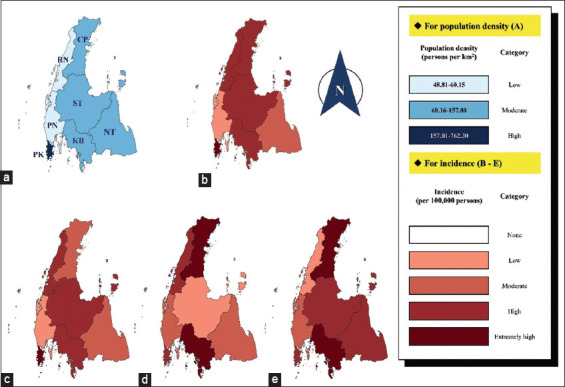
The spatial distribution of tuberculosis (TB) infected cases in the studied area including (a) Map of studied provinces and the density of the population, (b) Map of average TB incidence by area between 2020-2022, (c) Map of TB incidence by area in 2020, (d) map of TB incidence by area in 2021, and (e) Map of TB incidence by area in 2022. The map was generated using Quantum GIS version 3.34 software. (CP: Chumphon; RN: Ranong; ST: Surat Thani; PN: Phang Nga; KB: Krabi; NT: Nakhon Si Thammarat; PK: Phuket).

Regarding the spatial distribution of TB-infected cases, the incidence of TB was high in the province, which had a moderate to high density of population (Figures-4a and b). The highest average incidence of TB in this study was found in Phuket province (9.75/100,000 risk person-year). In contrast, Phang Nga province had the lowest average TB incidence (5.72/100,000 risk person-years). Mainly, the incidence of TB in this region was found to be high. The provinces with high incidence were Chumphon (7.78/100,000 risk person-year), Surat Thani (7.57/100,000 risk person-year), Krabi (8.08/100,000 risk person-year), and Phuket. The incidence of TB in each province was found to be different (Figures-4c-e). Interestingly, the incidences of TB in Chumphon, Krabi, and Nakhon Si Thammarat provinces were increasing; in contrast, the incidences in Ranong and Phang Nga provinces slightly decreased annually.

### Antibiotic resistance of clinical isolates of *M. tuberculosis*

The antibiotic resistance of the clinical isolates of *M. tuberculosis* was determined based on the first- and second-line anti-TB drugs. The results demonstrated that most clinically relevant *M. tuberculosis* isolates were resistant to isoniazid (Table-2). The detected isoniazid resistance was 394, 255, and 179 cases in 2020, 2021, and 2022, respectively. Resistance to rifampicin in 2020, 2021, and 2022 was 25, 9, and 12 cases, respectively. The results showed that 46, 24, and 29 isolates possessed multidrug resistance in the years 2020, 2021, and 2022, respectively. Pre-XDR and XDR of the clinically relevant *M. tuberculosis* isolates were detected during the time of this study. An increase in mono-resistance, MDR, pre-XDR, and XDR was observed in fiscal year 2022 compared with fiscal years 2020 and 2021.

**Table-2:** Antibiotic resistance of clinical isolates of *M. tuberculosis*.

The type of resistance	Antibiotics	Year		

		2020 (n = 2744) (%)	2021 (n = 1887) (%)	2022 (n = 670) (%)
Mono-resistance	Isoniazid	394 (14.36)	255 (13.51)	179 (26.72)
	Rifampicin	25 (0.91)	9 (0.48)	12 (1.79)
MDR	Isoniazid and rifampicin	46 (1.68)	24 (1.27)	29 (4.33)
Pre-XDR	Isoniazid, rifampicin, and fluoroquinolones^a^ or second-line injectables^b^	7 (0.26)	1 (0.05)	4 (0.60)
XDR	Isoniazid, rifampicin, fluoroquinolones, and second-line injectables	1 (0.04)	2 (0.11)	1 (0.15)

a Fluoroquinolone contain ofloxacin, levofloxacin, moxifloxacin.

b Second-line injectables containing amikacin, capreomycin, and kanamycin. *M. tuberculosis=Mycobacterium tuberculosis*, MDR=Multi-drug resistance, XDR=Extensively drug-resistant

### Detection of *inhA* and *katG* mutations

The detection of mutations in both *inhA* and *katG* genes associated with isoniazid resistance in *M. tuberculosis* was investigated. As detected by the line probe assay, 191 isolates showed an *inhA* mutation, whereas 248 isolates presented a *katG* gene mutation in the year 2020 (Table-3). In 2021, the detection of each *inhA* and *katG* mutation was 139 and 179 isolates, respectively. In addition, 43 samples with the *inhA* mutation and 55 isolates with the *katG* mutation were observed in the clinical isolates of *M. tuberculosis* collected in 2022. Furthermore, three isolates collected in 2020 and one isolate collected in 2021 possessed both *inhA* and *katG* mutations.

The detection of *inhA* and *katG* mutations associated with isoniazid resistance in *M. tuberculosis* is further determined by real-time PCR if the results of the line probe assay are invalid. As shown in Table-3, one isolate detected in 2020 showed both *inhA* and *katG* mutations. In 2021, three isolates possessed each *inhA* and *katG* mutation. It is interesting to note that 62 isolates were detected with the *inhA* mutation and 65 isolates with the *katG* mutation in 2022. Furthermore, one isolate showed both *inhA* and *katG* mutations.

**Table-3:** Detection of antibiotic-resistant genes in clinical samples of TB.

Methodology/year	No. of isolates carrying antibiotic-resistant genes		

	*inhA* alone	*katG* alone	*inhA* + *katG*
Line probe assay			
2020 (n = 442) (%)	191 (43.21)	248 (56.11)	3 (0.68)
2021 (n = 319) (%)	139 (43.57)	179 (56.11)	1 (0.31)
2022 (n = 98) (%)	43 (43.88)	55 (56.12)	0
Realtime PCR			
2020 (n = 3) (%)	1 (33.33)	1 (33.33)	1 (33.33)
2021 (n = 6) (%)	3 (50.00)	3 (50.00)	0
2022 (n = 128) (%)	62 (48.44)	65 (50.78)	1 (0.78)

TB=Tuberculosis, PCR=Polymerase chain reaction

## Discussion

TB, caused by *M. tuberculosis*, remains a significant global health concern. The present study revealed the occurrence of TB as well as the detection of multidrug resistance associated with *inhA* and *katG* genes in patients with *M. tuberculosis* in upper Southern Thailand. The data were provided by the Office of Disease Prevention and Control Region 11^st^, Nakhon Si Thammarat, Thailand, during the COVID-19 pandemic (2020–2022). The results revealed that most of the samples were obtained from patients located in Nakhon Si Thammarat province. This may be due to the location of the office in the province as well as the largest population of hospitals and people compared with other provinces in the upper Southern region of Thailand. However, the highest average incidence of TB in this study was in Phuket province, which may have affected the population density in the province. Interestingly, the incidences of TB in Chumphon province, Krabi province, and Nakhon Si Thammarat province increased by the year, whereas in Ranong province and Phang Nga province, they slightly decreased by the year. In 2018, 186 patients with TB were recorded and diagnosed at Sirindhorn Hospital in Bangkok, the capital city of Thailand [9]. It has been reported that 466/1002 (46.5%) participants had a latent TB infection, as detected in Klong Prem Central Prison, a maximum-security prison in Bangkok, Thailand [10]. At King Chulalongkorn Memorial Hospital in Thailand, there were 164 cases of TB/100,000 workers reported by Mingchay *et al*. [11]. Moreover, most of the patients with positive results were the young age group [11]. The incidence of TB in other Asian countries has been documented. Southern Asia (22%) showed the highest prevalence of TB, followed by Eastern Asia (18%) and South-east Asia (16%), as described by a systematic review and meta-analysis of articles published from 2012 to 2021 [12].

This study collected data on TB from 2020 to 2022, which was the period of the COVID-19 pandemic. The results revealed that the number of samples associated with TB in Upper Southern Thailand decreased over the study period. This finding was similar to the incidence of TB in several nations during the pandemic of COVID-19 [13]. Furthermore, the WHO has reported that the COVID-19 pandemic has negatively impacted TB case notifications compared with data from 2019 and 2020 [14]. However, our data indicated a rise of cases during COVID-19 peak times in 2020, which is 7565 cases, and in 2021 after COVID, it was reduced to 4511 cases. This may have resulted from human behavior during the COVID-19 pandemic. People stay home and wear a mask, which results in a reduction in direct contact with TB patients. In addition, Thai people still wear masks after COVID-19 spread.

Antibiotic combinations can be used to treat TB in humans. Isoniazid is one of the first drugs of choice for *M. tuberculosis* treatment because it inhibits mycolic acid biosynthesis. However, the occurrence of isoniazid-resistant *M. tuberculosis* caused by mutation *inhA* and *katG* has been reported in many countries, including Thailand [15, 16]. It is well known that the *inhA* gene encodes for enoyl acyl reductase and is involved in mycolic acid biosynthesis, whereas the *katG* gene encodes catalase-peroxidase, which is necessary for the activation of the prodrug isoniazid [7]. Therefore, the detection of the mutation of two genes in the clinical isolates may estimate isoniazid resistance in the area. According to the results, isoniazid was the most resistant antibiotic found in clinical *M. tuberculosis* isolates. This finding was similar to previous studies by Rudeeaneksin *et al*. [17] on the prevalence of antibiotic-resistant *M. tuberculosis* in Thailand and other countries like Indonesia [18]. It has been reported that the most prominent mutations of Ser315Thr substitution in *katG* and −15 C to T substitution in the regulatory region of *inhA* were detected in antibiotic-resistant *M. tuberculosis* in Thailand [17]. Depending on the *M. tuberculosis* population, different countries may have different mutation patterns and frequencies.

It was noticed that data analysis in this study was performed according to the standard procedure for TB recommended by the Office of Disease Prevention and Control Region, Thailand. Recently, the definitions of pre-extensively and XDR-TB have been updated by the WHO in 2021 [19]. However, the data collection and analysis of TB at the Office of Disease Prevention and Control Region 11^st^, Thailand, have been investigated using the old standard procedure available in Thailand [16]. This study revealed the detection of pre-XDR and MDR of clinically relevant *M. tuberculosis* isolates in the years 2020–2022. It was noticed that the percent resistance, including mono-resistance, MDR, pre-XDR, and XDR, of the clinically isolated *M. tuberculosis* isolates increased in 2022 compared with the previous years. Furthermore, six of the clinically relevant *M. tuberculosis* isolates possessed multidrug resistance, presenting both *inhA* and *katG* mutations during the years 2020–2022. This finding should be considered as a preventive and control strategy while treating patients with multidrug-resistant *M. tuberculosis*.

### Limitation

The limitation of this study was the collection of samples received from hospitals under the Office of Disease Prevention and Control Region 11^st^, Nakhon Si Thammarat, Thailand. It was noted that the private hospitals did not send the samples to the Office of Disease Prevention and Control Region 11^st^. Hence, the samples in this study were not all patients in Upper Southern Thailand.

## Conclusion

This study revealed a retrospective occurrence of TB and antibiotic-resistant *M. tuberculosis* isolated from patients with TB located in upper Southern Thailand during the years 2020–2022. The results showed that 51% of the samples were obtained from patients located in Nakhon Si Thammarat, followed by Surat Thani provinces. However, the highest incidence of TB in this study was found in Phuket province. Furthermore, 99 isolates were MDR, whereas four isolates were XDR. In addition, antimicrobial resistance associated with mutations in the *inhA* and *katG* genes was detected in the isolates. These findings contribute to the understanding of the occurrence of antibiotic-resistant TB that could lead to use as data for preventing MDR-TB.

## Authors’ Contributions

PK, PS, AB, and WM: Conceived and designed the study. PK, AB, SS, PW, and BB: Performed the study. PK, PS, AB, VN, MLP, and WM: Analyzed and interpreted the data. PK, PS, AB, and WM: Performed statistical analysis.PK, PS, VN, MLP, and WM: Wrote the manuscript. PK, PS, VN, MLP, and WM: Revised the manuscript. All authors have read, reviewed, and approved the final manuscript.
